# Smoking and alcohol, health-related quality of life and psychiatric comorbidities in Leber’s Hereditary Optic Neuropathy mutation carriers: a prospective cohort study

**DOI:** 10.1186/s13023-021-01724-5

**Published:** 2021-03-11

**Authors:** Andrea Rabenstein, Claudia B. Catarino, Verena Rampeltshammer, David Schindler, Constanze Gallenmüller, Claudia Priglinger, Oliver Pogarell, Tobias Rüther, Thomas Klopstock

**Affiliations:** 1 Department of Psychiatry and Psychotherapy, Ludwig-Maximilians University München, Nussbaumstr. 7, 80336 Munich, Germany; 2 Department of Neurology, Friedrich-Baur Institute, Ludwig-Maximilians University München, Ziemssenstr. 1a, 80336 Munich, Germany; 3 Department of Ophthalmology, Ludwig-Maximilians University München, 80336 Munich, Germany; 4 Munich Cluster for Systems Neurology (SyNergy), Munich, Germany; 5 German Center for Neurodegenerative Diseases (DZNE), Munich, Germany

**Keywords:** Smoking, Alcohol, Mitochondrial disorder, Depression, Leber’s hereditary optic atrophy, LHON, Quality of life, Mental health (< 10)

## Abstract

**Background:**

Leber’s hereditary optic neuropathy (LHON) is a rare mitochondrial disorder, characterized by acute or subacute bilateral vision loss, frequently leading to significant chronic disability, mainly in young people. The causal LHON mutations of the mitochondrial DNA have incomplete penetrance, with the highest risk of disease manifestation for male mutation carriers in the second and third decades of life. Here we evaluated smoking, alcohol drinking habits, health-related quality of life (QOL) and psychiatric comorbidities in a cohort of LHON patients and asymptomatic mutation carriers from a tertiary referral centre.

**Methods:**

Cross-sectional analysis of the ongoing Munich LHON prospective cohort study. Participants included all LHON patients and asymptomatic LHON mutation carriers older than 16 years at baseline, who were recruited between February 2014 and June 2015 and consented to participate. General, neurological and ophthalmological investigations were performed, including validated questionnaires on smoking, alcohol drinking habits, depressive symptoms and health-related QOL.

**Results:**

Seventy-one participants were included, 34 LHON patients (82% male) and 37 asymptomatic mutation carriers (19% male). Median age at baseline was 36 years (range 18–75 years). For LHON patients, median age at visual loss onset was 27 years (9 to 72 years). Smoking is more frequent in LHON patients than asymptomatic LHON mutation carriers, and significantly more frequent in both groups than in the general population. Sixty percent of LHON patients, who smoked at disease onset, stopped or significantly reduced smoking after visual loss onset, yet 40% of LHON patients continued to smoke at study baseline. Excessive alcohol consumption is more frequent in male LHON patients than in LHON asymptomatic and more frequent than in the male general population. Further, female asymptomatic LHON mutation carriers are at risk for depression and worse mental QOL scores.

**Conclusions:**

Given the high prevalence of smoking and excessive drinking in LHON mutation carriers, implementing effective measures to reduce these risk factors may have a significant impact in reducing LHON disease conversion risk. The underrecognized prevalence of mental health issues in this population of LHON mutation carriers highlights the need for awareness and more timely diagnosis, which may lead to improved outcomes.

**Supplementary Information:**

The online version contains supplementary material available at 10.1186/s13023-021-01724-5.

## Background

Leber’s Hereditary Optic Neuropathy (LHON, OMIM #535000) is one of the most frequent mitochondrial disorders, with an estimated prevalence of 1:31,000 [1]. LHON leads to subacute bilateral vision loss, progressing within weeks or months, often resulting in significant chronic visual disability. Spontaneous recovery may occur, yet long-term visual outcome is often poor. Onset of visual loss is most frequent in male adults in the second or third decades of life [2], although female patients, childhood-onset cases [3, 4] and late-onset cases [5] may be underdiagnosed. More than 90% of LHON is caused by one of three frequent point mutations of the mitochondrial DNA (mtDNA), m.11778G > A [6], m.3460G > A [7] or m.14484T > C [8], in genes coding subunits of complex I of the respiratory chain of the mitochondria [2]. Dysfunction of complex I leads to reduced adenosine triphosphate (ATP) production [9], increased reactive oxygen species, which cause dysfunction of the retinal ganglion cells (RGC), which are particularly susceptible to mitochondrial dysfunction, and later cause apoptosis of a proportion of the RGC [10]. LHON follows maternal inheritance with incomplete penetrance. There is a substantial differential risk between genders, as 50% of male but only 10% of female LHON mutation carriers may develop visual loss due to LHON in their lifetime [2]. This suggests a role for other genetic [11, 12] and environmental modifiers [13–15]. Environmental factors add to the genetic risk and some are modifiable, such as smoking and excessive alcohol consumption [11]. Epidemiological studies showed smoking is a risk factor for a mutation carrier to develop LHON symptoms [11]. An association between smoking and decreased complex I activity [16] suggests a mechanism for how smoking may aggravate genetically-determined mitochondrial dysfunction in LHON. Indeed, the risk of disease for male LHON mutation carriers was shown to increase from 50 to 93% in smokers in a large epidemiological study [11, 17]. Heavy alcohol intake has also been associated with a trend for increased risk of disease in LHON carriers [11]. Further, it has been shown that onset of visual loss negatively impact the quality of life of LHON mutation carriers [18]. A systematic analysis of health-related QOL and psychiatric comorbidities in a population of LHON patients and asymptomatic LHON mutation carriers has not yet been reported. With this study, we aimed to quantify the modifiable environmental risk factors, particularly smoking behaviour and alcohol consumption habits, as well as depressive symptoms and health-related QOL, for both LHON patients and asymptomatic LHON mutation carriers in a large well-characterized cohort.

## Methods

### Participants; LHON patients and asymptomatic LHON mutation carriers

Of 81 participants recruited from February 2014 to June 2015 in the Munich LHON cohort study, 71 were 16 years and older at baseline and were included in the final analysis. Thirty-four (48%) were LHON patients with a known pathogenic LHON mutation of the mtDNA and 37 (52%) were asymptomatic LHON mutation carriers. All participants and legal guardians (for participants under the age of 18) gave written informed consent or assent. The local Ethics committee of the Ludwig-Maximilians-University (LMU) of Munich approved this project (project number 278–13).

### Study design

Participants in the Munich LHON cohort study are examined at baseline and thereafter annually by an expert team of neurologists, ophthalmologists and psychiatrists in the outpatient clinics of the LMU University Hospital. Each visit includes thorough medical history, family history, as well as systematic general, neurological and ophthalmological examinations, and laboratory examinations. Validated questionnaires on smoking habits and alcohol consumption, quality of life and psychiatric comorbidities are completed at each visit. All subjects are advised at each visit to quit smoking, moderate alcohol intake and keep a balanced diet. Information on smoking cessation programmes is provided to all participants, who smoke.

### Smoking-related data

The accuracy of self-reported smoking status was checked by determining the concentration of carbon monoxide in expired air, measured with a calibrated smoking meter BMC 2000 (SNEKO, Osan). Ninety-six percent of participants, who smoked at baseline, completed the Fagerström Test for Nicotine Dependence (FTND), which measures the degree of nicotine dependence [19, 20]. Smoking was quantified in pack-years. A current smoker was defined if the participant smoked in the last 30 days; or ex-smoker, if they smoked more than 100 times but not in the last 30 days; and non-smoker, if they smoked in total less than 100 times in life [21]. The proportion of smokers, for both LHON patients and asymptomatic LHON mutation carriers, was compared to the prevalence in the general population in Germany, stratified by gender [22].

### Alcohol consumption data

The Alcohol Use Disorders Identification Test (AUDIT) questionnaire for detection of excessive alcohol consumption [23], which includes an estimation of number of days with alcohol consumption in the previous 30 days, and type and number of alcoholic beverages in an average day of alcohol consumption, was completed by 93% (66/71) of the participants. Total amount and average daily alcohol consumption were calculated for each participant. The alcohol-by-volume (ABV) measures used were 4.8% for beer, 11.0% for wine/sparkling wine and 33.0% for liquor [24]. For both groups, a quantity-frequency-index was calculated as a measure of alcohol consumption. For all participants this was calculated at study baseline; and, for LHON patients, also for the six months preceding onset of visual loss. Excessive daily alcohol consumption was defined as more than 12 g/day for females, and 24 g/day for male participants [25]. Seventy-nine percent of LHON patients and 73% of asymptomatic LHON mutation carriers answered the question on estimated daily amount of alcohol consumption at study baseline.

### Data collection on depressive symptoms

The Beck Depression Inventory (BDI-I) is a 21-item self-report measure of depressive symptoms [26], which was completed by 88% (30/34) of LHON patients and 89% (33/37) of asymptomatic LHON mutation carriers. Cut-off values were used as recommended by the current German S3/ National health policy guidelines on depression [27]. The frequency of depression in both LHON patients and in LHON asymptomatic mutation carriers was compared with the prevalence rate of depression in the general population, which currently is 16–20% [28, 29], with the frequency of depressive symptoms estimated at 10.2% for females and 6.1% for males in the German population [30].

### Health-related quality of life (QOL)

Health-related QOL was assessed using the SF-12 version 2 (SF-12 v2) questionnaire [31]. Ninety-six percent (68/71) of participants completed the SF-12 v2 questionnaire. Two sub-scores were calculated, the Physical Component Summary (PCS) and the Mental Component Summary (MCS). Both sub-scores were compared with normative data for the general population, stratified by gender [32].

### Statistical analysis

Nominal data were compared by Chi-square tests and Fisher’s exact tests. Continuous data were compared by independent sample t-tests and one sample t-tests. As gender distribution was significantly different between LHON patients and asymptomatic LHON mutation carriers in our study, the statistical analyses were stratified by gender. Statistical analyses were performed using SPSS version 25.0 for Windows (Armonk, NY:IBM Corp., 2018). A *p* value of less than 0.05 was considered statistically significant.

## Results

The demographic characteristics of the 71 study participants are summarized in Table 1.

**Table 1:** Demographic characteristics and type of mutation of the participants in the present study

	LHON patients (n = 34)	Asymptomatic LHON mutation carriers (n = 37)	Total (n = 71)
*Gender, n (%)*			
M	28 (82%)	7 (19%)	35 (49%)
F	6 (18%) *	30 (81%)*	36 (51%)
M:F ratio	4.7:1	1:4.3	N/A
*Age at study baseline (y)*			
Mean (± SD)	38 (± 14)	42 (± 13)	40 (± 14)
Median (range)	36 (18–75)	43 (18–70)	39 (18–75)
*Age at clinical onset (y)*			
Mean (± SD)	31 (± 15)	N/A	N/A
Median (range)	27 (9–72)	N/A	N/A
*Mutation, n (%)*			
m.11778G > A	23 (68%)	28 (76%)	51 (72%)
m.3460G > A	6 (18%)	4 (11%)	10 (14%)
m.14484 T > C	4 (12%)	3 (8%)	7 (10%)
Others^#^	1 (2%)	2 (5%)	3 (4%)

F, female; M, male; N/A, not applicable; SD, standard deviation; y, years; # rare causal LHON mutation: m.14487 T > C (one participant) [33]; m.3958G > A (two participants). *p < 0.05

Of 34 LHON patients, 28 (82%) were male (male:female ratio 4.7:1). Median age at study baseline was 36 years (range 18–75 years). Median age at clinical onset was 27 years (range 9–72 years). Median duration of disease at study baseline was 2.5 years (range 4 months—37 years). Twenty-five LHON patients (74%) were treated with idebenone 900 mg/day.

Of 37 asymptomatic LHON mutation carriers, seven (19%) were male (male:female ratio 1:4.3), with median age at baseline 43 years (range 18–70 years). The gender distribution was significantly different between LHON patients and asymptomatic mutation carriers. Most asymptomatic LHON mutation carriers (94%) are related to LHON patients also included in the study.

Sixty-eight participants (96%) had one of the three primary LHON mutations of the mtDNA, and only three had a rare causal LHON mutation, one patient had the mutation m.14487 T > C [33] in the *ND6* gene (n = 1) while one patient and one asymptomatic mutation carrier had the mutation m.3958G > A [34] in the *ND1* gene. Vitamin B12 deficiency was measured at baseline or documented in the previous medical history in 44% of the LHON patients in our cohort.

### Smoking behaviour

The distribution of self-reported smoking behaviour for all participants is shown in Table 2. Data on smoking habits was available for LHON patients for the six months before onset of vision loss and also at study baseline, while for asymptomatic LHON mutation carriers smoking data was available only at study baseline, which here is equivalent to “before onset”, because per definition there was no visual loss onset in the asymptomatic group.

**Table 2:** Information on smoking behaviour at study baseline

	LHON patients at baseline (n = 34)	LHON patients before onset (n = 34)	Asymptomatic LHON mutation carriers (n = 37)	General population
*Smoking status, n (%)*				
Ever-smokers	26 (76%)	23 (68%)	15 (41%)	
M	22/28 (79%)	19/28 (68%)	5/7 (71%)	
F	4/6 (67%)	4/6 (67%)	10/30 (33%)	
Current smokers	16 (42%)	20 (59%)	12 (32%)	25%
M	14/28 (50%)	17/28 (61%)	5/7 (71%)	30%
F	2/6 (33%)	3/6 (50%)	7/30 (23%)	20%
Ex-smokers	10 (29%)	3 (9%)	3 (8%)	
M	7/28 (25%)	2/28 (7%)	0/7 (0%)	
F	3/6 (50%)	1/6 (12%)	3/30 (10%)	
Never smokers	8 (24%)	11 (32%)	22 (59%)	
M	6/28 (21%)	9/28 (32%)	2/7 (29%)	
F	2/6 (33%)	2/6 (33%)	20/30 (67%)	
*Pack-years, for ever-smokers*				
Mean (± SD)	14.1 (± 13.0)		21.1 (± 14.5)	
Range	0–42		0.1–102	
*FTND, for current smokers Nicotine dependence:*				
Very high	2 (15%)		0 (0%)	
High	1 (8%)		4 (36%)	
Moderate	2 (15%)		2 (18%)	
Low	8 (62%)		5 (45%)	

FTND, Fagerström Test for Nicotine Dependence; SD, standard deviation

The concentration of carbon monoxide in expired air correlated significantly with the self-reported daily cigarette consumption (*p* < 0.001).

In our cohort, the proportion of ever-smokers was higher among LHON patients at onset than in asymptomatic LHON mutation carriers (68% vs. 41%), and for each subgroup higher than in the general population (25%; *p* = 0.02).

Before symptom onset, 59% (20/34) of LHON patients and 42% (16/37) of asymptomatic LHON mutation carriers were current smokers, while 32% (11/34) of LHON patients at onset and 59% (22/37) of asymptomatic LHON mutation carriers were never-smokers.

For male participants, 61% (17/28) LHON patients before onset and 71% (5/7) asymptomatic mutation carriers were current smokers, which is, for both groups, significantly more than the frequency of smokers in the general male population in Germany (30%; *p* = 1 × 10^–5^ and *p* = 0.017, respectively) [22] (Additional file 1: Figure S1).

For female participants, 50% (3/6) LHON patients before onset and 23% asymptomatic LHON mutation carriers were current smokers, not significantly different to the general female population (20%) [22].

**Fig. 1:**
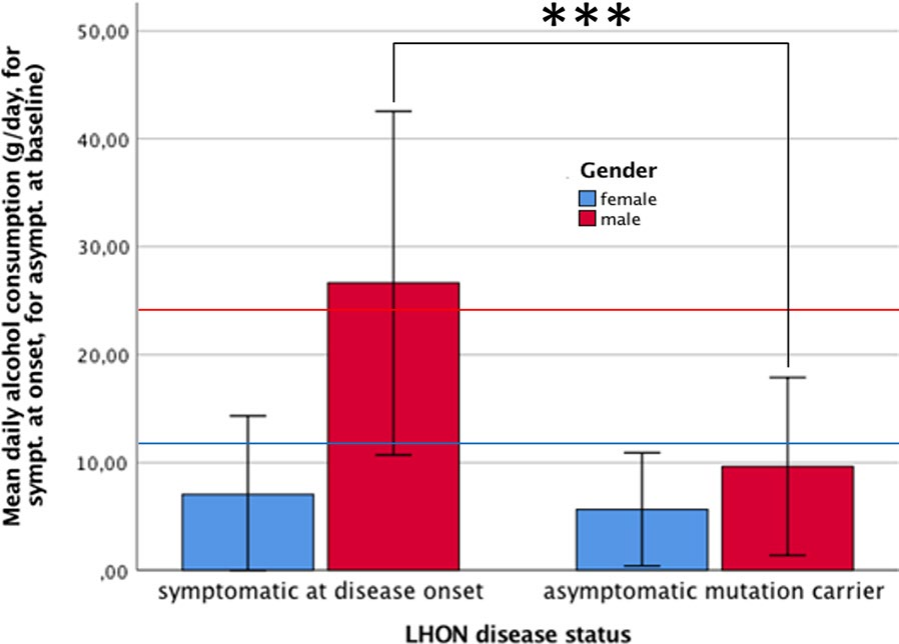
Daily alcohol consumption before symptom onset, for LHON patients, and at baseline, for asymptomatic carriers. Average daily alcohol consumption (in g/day) in the six months before visual loss onset, for LHON patients, and at study baseline, for LHON asymptomatic mutation carriers. The threshold for excessive alcohol consumption in the general population is represented in blue for females (9 g/day) and red for males (16 g/day)

**Fig. 2:**
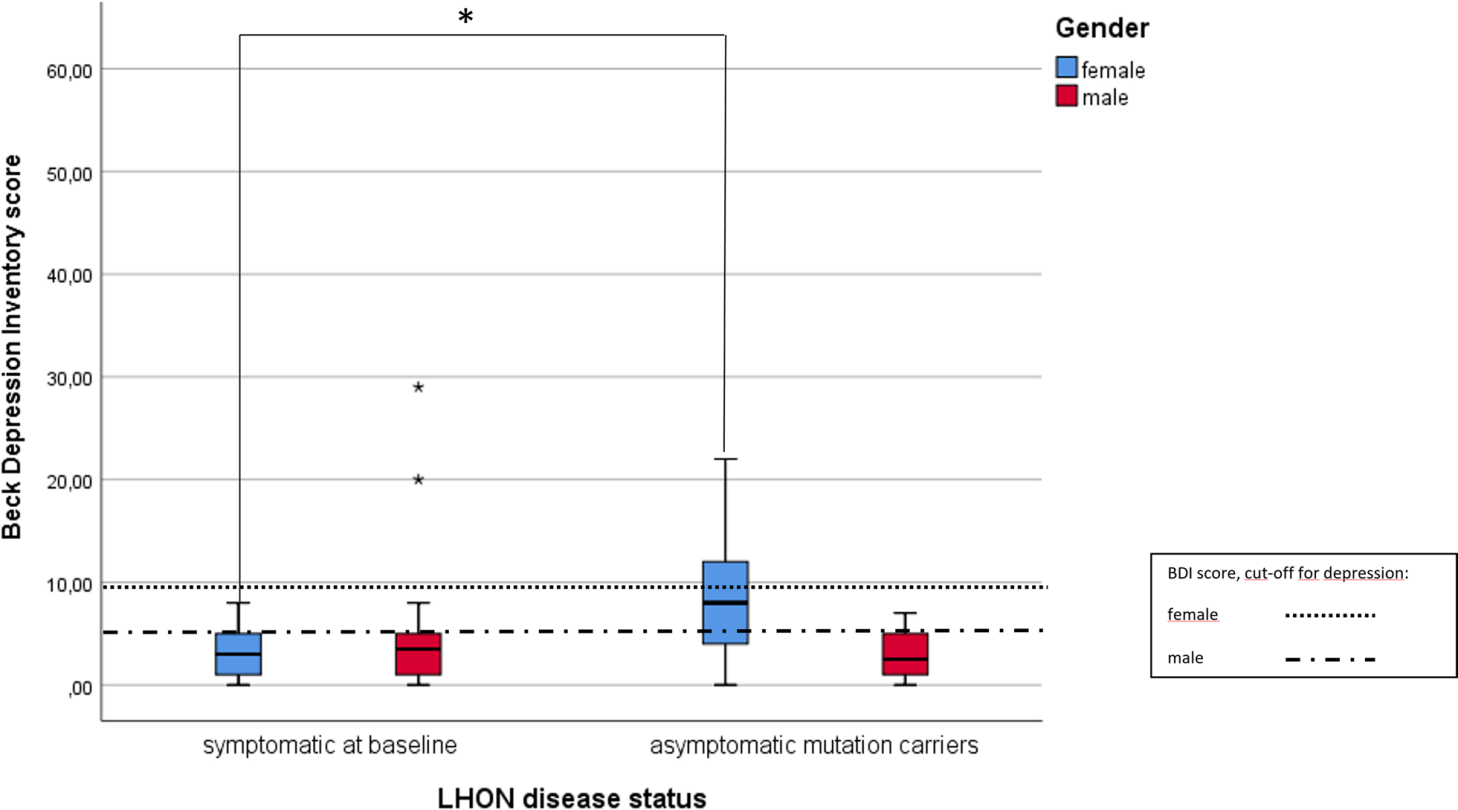
Depression data for LHON patients and asymptomatic carriers, as assessed by the BDI-I questionnaire. Box plots of depression data, as given by the BDI-I score, for LHON patients and asymptomatic LHON mutation carriers, stratified by gender. The whiskers represent the 95% CI. The thresholds in the general population are represented for females and males

Additional file 2: Figure S2 shows a flowchart of smoking behaviour over time for the LHON patients, before disease onset and at study baseline. While very few LHON patients started smoking after symptom onset and five LHON patients continued their smoking behaviour despite visual loss onset, 60% (12/20) of LHON patients, who had smoked at disease onset, either stopped smoking or reduced smoking significantly after onset of visual loss. Interestingly, for about 45% of the LHON patients, who stopped smoking, some improvement of the visual acuity could be documented at last follow-up, versus 41% of the LHON patients, who continued smoking as before onset, but the numbers are not sufficiently large for robust conclusions (Figs. 1, 2).

The FTND score, which measures the degree of nicotine dependence, showed no statistically significant difference between LHON patients and asymptomatic LHON carriers (Table 2).

### Alcohol consumption

Data on alcohol consumption in our cohort is summarized in Table 3, for both LHON patients and asymptomatic LHON mutations carriers, stratified by gender.

**Table 3:** Information on self-reported alcohol consumption in our cohort of LHON mutation carriers, at study baseline

	LHON patients (n = 34)	Asymptomatic LHON mutation carriers (n = 37)	General population
*Self-reported excessive alcohol consumption before onset*			
(27/34 with data: 21 M/6F)	8 (30%)	N/A	14%
M	7 (33%)	N/A	M 16%
F	1 (17%)	N/A	F 13%
*Self-reported excessive alcohol consumption at study baseline*			
(64/71 with data)	7 (26%)	2 (6%)	14%
M	5 (24%)	0 (0%)	M 16%
F	2 (33%)	2 (7%)	F 13%
AUDIT questionnaire, n (%)			
Excessive alcohol consumption at study baseline (66/71 with data:32 Pat./34 Asymp.)	6 (19%)	4 (12%)	
*Daily alcohol consumption at study baseline, g/day (68/71 with data: 33 Pat./35 Asymp.)*			
Mean (± SD)	12.8 (± 15.8)	6.4 (± 11.1)	
Range	0–60	0–54	
*Days of alcohol consumption at study baseline, n (%) (68/71 with data: 33 Pat./35 Asymp.)*			
Daily	3 (9.1%)	1 (2.9%)	
Weekly	12 (36.4%)	10 (28.6%)	
Seldom	11 (33.3%)	19 (54.3%)	
Non-drinker	7 (21.2%)	5 (14.3%)	

Asymp., asymptomatic mutation carriers; AUDIT, Alcohol Use Disorders Identification Test; Pat., patients; SD, standard deviation

Thirty percent (8/27) LHON patients, who filled out the questionnaires on alcohol consumption, reported having had excessive alcohol consumption at disease onset [25]. Some LHON patients reported having reduced the alcohol consumption after disease onset, but the proportion of male LHON mutation carriers with excessive alcohol consumption (26%) at study baseline was still significantly higher among LHON patients than in asymptomatic LHON mutation carriers (*p* < 0.001). None of the five male asymptomatic LHON mutation carriers reported excessive alcohol consumption. There is a higher proportion of male LHON patients (30%) drinking alcohol excessively, compared to the male general population in Germany (15.6%) [21, 25]. This was confirmed by the AUDIT data, where 19% of LHON patients presented excessive alcohol consumption even at study baseline, which was not the case for asymptomatic mutation carriers, as shown in Fig. 1.

### Depression data

#### Increased depression rates in female asymptomatic LHON mutation carriers

For female asymptomatic LHON mutation carriers, 44% (12/27) had mild to moderate depression, which is higher than the prevalence rate of depression in the German female general population of 10.2% (28–30). No male asymptomatic LHON mutation carrier in this small subgroup (*n* = 7) reported depressive symptoms at study baseline (Table 4).

**Table 4:** Information on health-related quality of life (QOL) and prevalence of depressive symptoms at study baseline

	LHON patients (n = 34)	Asymptomatic LHON mutation carriers (n = 37)	General population
*SF-12 v2 at study baseline*			
(68/71 with data)	33/34	35/37	
MCS	49.5 ± 10.3	41.4 ± 9.5	N/A
M	48.7 (± 10.6)	50.38 (± 7.0)	50.4 (± 9.9)
F	53.3 (± 8.9)	39.59 (± 8.9)*	48.4 (± 9.6)
PCS	46.7 ± 6.9	54.3 ± 9.6	N/A
M	47.3 (± 6.6)	57.1 (± 3.5)*	50.6 (± 10.2)
F	43.9 (± 8.4)	53.7 (± 10.4)	48.7 (± 9.6)
*BDI at study baseline*			
(63/71 with data)	30/34	33/37	
*Depressive symptoms:*			
Severe	0	0	(Depression 16–20%;10% F; 6% M))
Moderate	2 (7%) [2M]	2 (6%) [2F]	
Mild	0	10 (30%) [10F]	
Depression score	4.5 ± 6.0	8.1 ± 6.0*	

BDI, Beck Depression Inventory; F, female; M, male; MCS, Mental Component Summary; N/A, not available; PCS, Physical Component Summary; SD, standard deviation; SF-12 v2, SF-12 version 2 questionnaire

*p < 0.05

Female LHON mutation carriers at study baseline showed Beck Depression Inventory (BDI-I) depression scores, which were significantly higher for asymptomatic LHON carriers than for LHON patients (*p* = 0.02), as shown in Fig. 2.

For male LHON patients at study baseline, 8% (2/24) had mild to moderate depression, not significantly different to the 6% frequency of the male general population. In the small subgroup of female LHON patients (*n* = 6), none had criteria for depression at study baseline, but we could not collect the more relevant data on depression at LHON symptom onset in our study.

### Lower mental health-related QOL in female asymptomatic LHON mutation carriers

Table 4 summarizes the health-related QOL data for our cohort of LHON patients and asymptomatic LHON mutation carriers, stratified by gender, as given by the SF-12v2. Mean PCS score was significantly lower for male LHON patients than the male general population (*p* = 0.015). PCS scores were also lower in the female LHON patients than in the female general population, but this was not statistically significant. For asymptomatic LHON mutation carriers, mean PCS scores were significantly higher for males (*p* = 0.006) and females (*p* = 0.016), compared to the gender-matched general population.

**Fig. 3:**
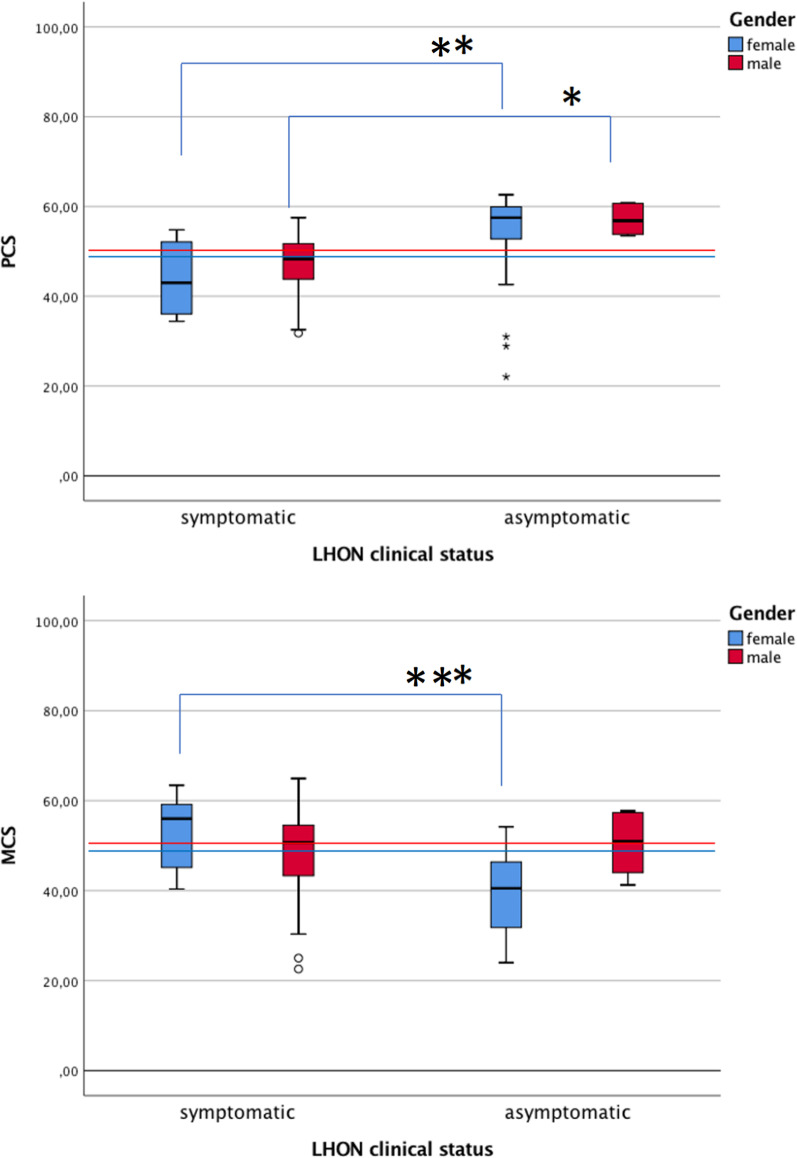
Health-related quality of life for LHON patients and asymptomatic carriers, assessed by SF-12 v2 questionnaire. Box plots of the SF-12 v2 sub-scores: Physical Component Summary (PCS, above) and Mental Component Summary (MCS, below). The whiskers represent the 95% CI. The thresholds in the general population are represented in blue for females (MCS 48.4 and PCS 48.7) and red for males (MCS 50.4 and PCS 50.6)

Remarkably, the MCS score, which is a proxy for mental health QOL, was lower for asymptomatic LHON mutation carriers than the general population (*p* < 0.001), and lower than for LHON patients at study baseline (*p* = 0.001) (Fig. 3). Interestingly, we found that female asymptomatic LHON mutation carriers score worse in mental QOL (MCS score) when compared to the female general population (*p* < 0.001). For the subgroup of female LHON patients, the sample is too small for robust comparisons. For males, there were no significant differences of the MCS scores, both for symptomatic and asymptomatic groups, compared to the general population.

## Discussion

LHON is the most frequent mitochondrial disorder and causes a high socio-economic burden. With the present study, we were able to show that LHON also causes a significant burden in mental health and quality of life, not only for LHON patients, but also for the asymptomatic at-risk relatives, who are carriers of a causal LHON mutation without showing symptoms.

Further, we could confirm the relevance of the previously described association between LHON and smoking as a potential risk factor for LHON mutation carriers [11], suggesting there is potential for risk reduction by prophylactic interventions such as lifestyle changes in this population. Both LHON patients and asymptomatic carriers smoke significantly more than the general population, with increased exposure to smoking described in large epidemiological studies to increase risk to develop disease. Smoking exposure for asymptomatic LHON mutation carriers may increase their risk of developing LHON, even if at the time of the study the disease may have not yet manifested.

Onset of visual loss in a previously asymptomatic LHON mutation carrier may serve as motivation to rethink smoking. Indeed, our data show that 60% of LHON patients, who were smokers at symptom onset, changed their smoking behaviour after experiencing onset of visual loss, with about a third stopping smoking altogether after disease onset. We found that 45% of the LHON patients, who stopped smoking, showed some improvement of visual acuity at last follow-up, compared to 41% of patients, who continued smoking despite onset of visual loss. However, numbers are relatively small. Also, the percentage of idebenone intake was different between the groups with clinical improvement (93%) and without clinical improvement (65%), and other factors may have confounded this analysis, which warrants analysis in a larger cohort.

Sharing the diagnosis of LHON with the patient and the relatives, in combination with effective communication of the relevance of the modifiable risk factors, presents a valuable opportunity to promote smoking cessation, with every subsequent visit in clinic providing a renewed chance to readdress this with the patient. At diagnosis, the LHON patient may be more motivated to quit smoking, as has been previously shown in cancer and chronic obstructive pulmonary disease [35, 36]. Remarkably, one-third of LHON patients in our study will keep on smoking. Information on the health risks of smoking, but also advice and support on quitting addictive behaviours should be provided to LHON patients and asymptomatic mutation carriers at every clinic visit.

Concordant with the previously described association between heavy alcohol intake and increased risk of LHON [11], our study showed a statistically significant higher proportion of male LHON mutation carriers with excessive alcohol consumption at disease onset, compared to the male general population [21]. A proportion of LHON patients stopped or reduced their drinking habits after visual loss onset, but the prevalence of excessive alcohol consumption at study baseline was still more frequent than the prevalence of the general population.

Further, vitamin B12 deficiency was documented, either in the laboratory tests at baseline or in the previous medical history, in a significant proportion of the LHON patients in our cohort. A vitamin B12 deficiency may play a modulating role in the development of optic neuropathy, can be easily diagnosed and treated, and should therefore be routinely looked for in the population of LHON mutation carriers. This warrants further research in a larger cohort.

To our knowledge, ours is the first study looking at mental health and physical health-related QOL in LHON mutation carriers. It had previously been shown that visual deficits in LHON negatively impact the visual-related QOL of LHON patients, as measured by the VF-14 scale [18, 37], but no study had previously systematically looked at the mental and physical health-related domains of QOL in the population of LHON mutation carriers. There are many dimensions to how the physical and mental QOL may be impacted in a LHON mutation carrier, given the risk of developing severe chronic visual impairment, or, for female carriers, also the risk their children may develop visual loss. In this study, we were able to show that female asymptomatic LHON mutation carriers score worse in mental QOL than the general population.

Further, the psychological impact of having a LHON mutation or becoming a patient with LHON had previously not been systematically studied. It has been described in the literature that men are, in general, more likely to develop or worsen addictive behaviours, such as alcoholism, whereas women are more likely to develop depressive symptoms, and that mental health and health-related behaviours, such as smoking and alcohol consumption, are closely linked [30, 38]. In the subgroup of LHON mutation carriers we have observed this, with male LHON patients and male asymptomatic mutation carriers smoking significantly more than the general population. Also, our study highlights a high frequency of mental health issues in female asymptomatic LHON mutation carriers, with the mental well-being of female asymptomatic mutation carriers shown to be worse than the female general population. Further, we found that mild and moderate depression is more frequent in female asymptomatic mutation carriers. It can be expected that screening the mental health of this population may allow earlier identification and treatment of mental health issues. These may be compounded by the stress of having an affected relative, uncertainty given the risk of developing the disease, with consequent vision loss and disability, as well as uncertainty given the risk for their children. Our findings on QOL and on depression are consistent, for LHON patients and asymptomatic mutation carriers. Depressive symptoms are known to have a negative impact on health-related QOL [39]. Enhancing individual health-related QOL is an important therapeutic goal of every treatment and this is relevant when treating and counselling both LHON patients and asymptomatic LHON mutation carriers. In general, it has been described that people with psychiatric comorbidities, especially those with depressive symptoms, are more likely to be non-compliant to medical recommendations [40] and poor mental health is associated with health-related behaviours such as smoking, physical inactivity and poor nutrition [41]. In our study we did not look systematically at nutrition, physical activity and compliance to medical recommendations, for LHON mutation carriers.

Our study has some limitations. The data we collected on depression and QOL for the LHON patients reports to the study baseline, which occurred from 4 months to 36 years after onset of visual loss, which may mean that at least a proportion of LHON patients may already have readjusted [18], and, therefore, we may underestimate the real impact of depression and mental health issues in LHON patients. There is significant phenotypical heterogeneity in LHON and a large spectrum of disease severity and disability, with a proportion of patients having significant clinical improvement, others no clinical improvement whatsoever and severe visual loss, which may influence QOL and psychological impact of disease. LHON patients would be expected to have higher depression scores than what we found, if the questionnaires had been administered sooner after symptom onset, when they first start experiencing visual loss or shortly thereafter. After several months or years, some LHON patients may already had time to adapt to the new condition, therefore this analysis performed at study baseline may underestimate the frequency of depression in the LHON patients. Further, the data on alcohol consumption was self-reported, which may have yielded underreporting for some patients. Another limitation of our study is that published data on the general population was used for comparison, given the small size of our group of healthy controls, which did not allow robust statistical comparisons. Although our LHON cohort study has been set up longitudinally, the current study is based on the cross-sectional analysis of the baseline data of the subjects enrolled in the first two years. We cannot know at study baseline whether and when any asymptomatic mutation carrier will develop LHON disease in the future. Further, some recall bias can be expected for the retrospective self-reported data on smoking and alcohol consumption before clinical onset of LHON, making the information on exposure to environmental triggers before clinical onset, its duration and quantification, less accurate, even more so for LHON patients with longer disease duration.

## Conclusions

Our study provides insights on lifestyle factors, psychiatric comorbidities, health-related QOL and psychological impact in both LHON patients and asymptomatic LHON mutation carriers. The high prevalence of smoking in LHON mutation carriers and excessive alcohol consumption in male LHON mutation carriers emphasizes the chance for effective prophylactic measures in the population of LHON mutation carriers, which may have a significant impact on future risk of disease manifestation and prognosis. We could show that female asymptomatic LHON mutation carriers are at particular risk for mental health issues. Promoting mental health in LHON mutation carriers may be expected to lead to more timely treatment and have a positive impact in mental health and QOL in LHON mutation carriers.

## Supplementary Information

**Additional file 1**: **Figure S1** Flow chart describing smoking behaviour for the LHON patients in our cohort over time. Smoking behaviour before disease onset and at study baseline.

**Additional file 2**: **Figure S2** Smoking habits for all participants in the study, LHON patients and asymptomatic LHON mutation carriers.

## Data Availability

The data analyzed during this study are available from the corresponding authors on reasonable request.

## Abbreviations

ABV: Alcohol-by-volume
AUDIT: Alcohol use disorders identification test
BDI: Beck depression inventory
ETDRS: Early treatment diabetic retinopathy study
FTND: Fagerström test for nicotine dependence
LHON: Leber’s hereditary optic neuropathy
MCS: Mental component summary
mtDNA: Mitochondrial DNA
PCS: Physical component summary
QOL: Quality of life
