# Supplementary figures and images for: Smoking and alcohol, health-related quality of life and psychiatric comorbidities in Leber’s Hereditary Optic Neuropathy mutation carriers: a prospective cohort study
Source: Orphanet J Rare Dis. 2021 Mar 11;16:127. doi: 10.1186/s13023-021-01724-5 (PMC7953635; doi:10.1186/s13023-021-01724-5)

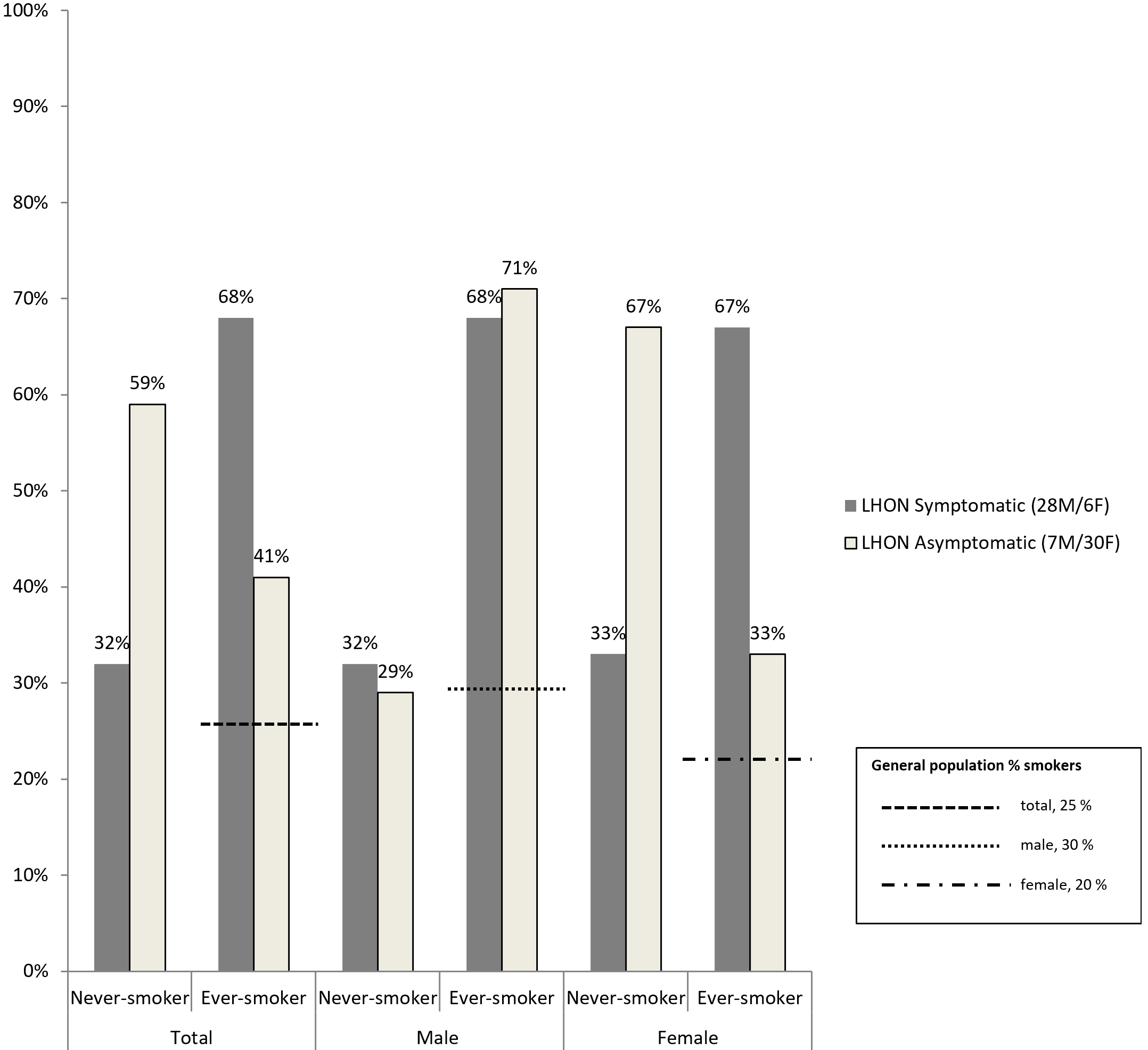

Supplement: Supplementary file 1 — Additional file 1: Figure S1 Flow chart describing smoking behaviour for the LHON patients in our cohort over time. Smoking behaviour before disease onset and at study baseline. [file 13023_2021_1724_MOESM1_ESM.tif]

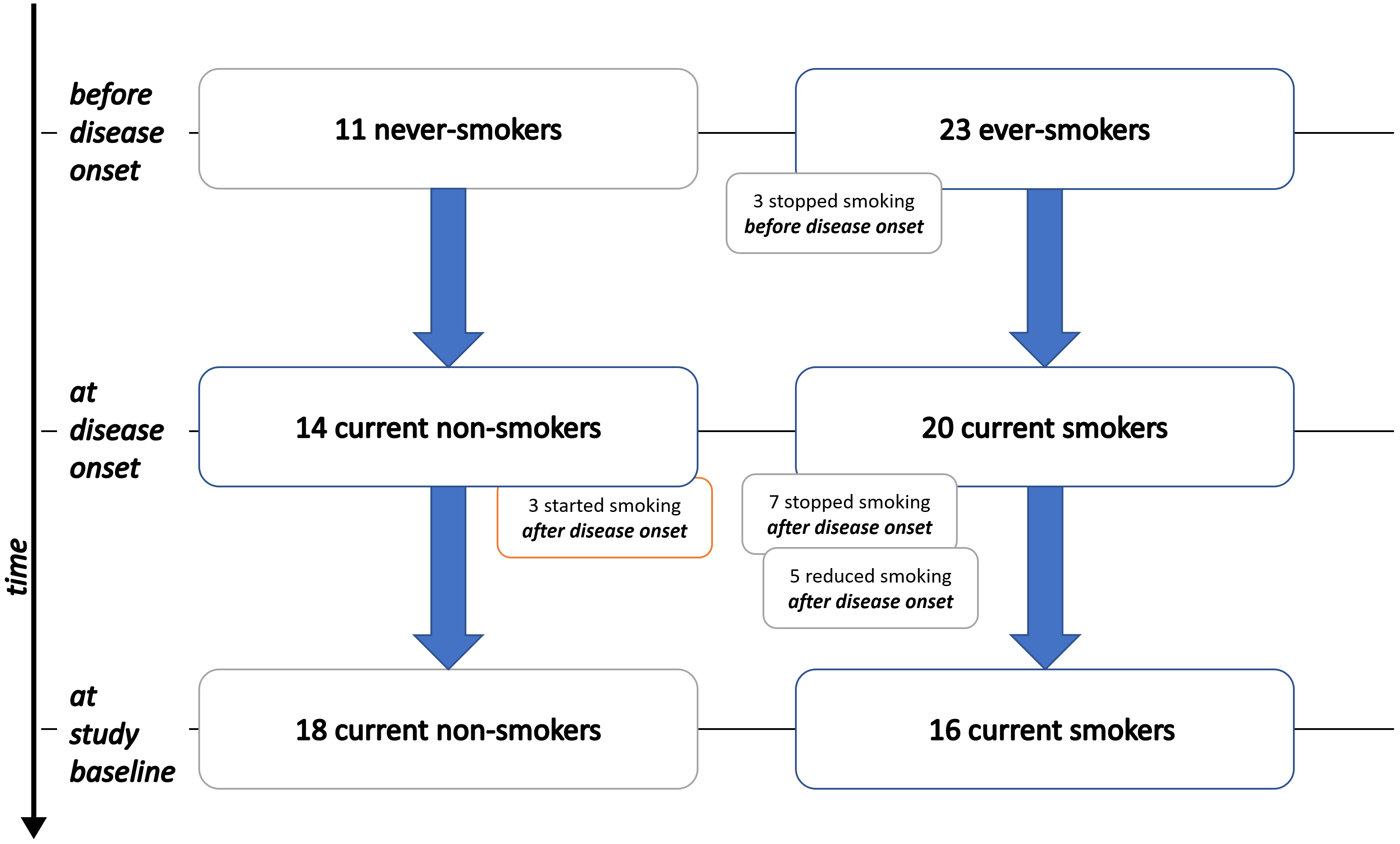

Supplement: Supplementary file 2 — Additional file 2: Figure S2 Smoking habits for all participants in the study, LHON patients and asymptomatic LHON mutation carriers. [file 13023_2021_1724_MOESM2_ESM.tif]
